# The role of cardiac computed tomography in diagnostic and prognostic assessment of pregnancy related spontaneous coronary artery dissection: a case report

**DOI:** 10.1093/omcr/omae030

**Published:** 2024-04-25

**Authors:** Alessandra Luciano, Cecilia Cerimele, Daniele Mecchia, Marcello Mozzani, Simone Steffani, Francesca D’Errico, Carlo Di Donna, Vincenzo De Stasio, Francesco Garaci, Marcello Chiocchi

**Affiliations:** Department of Biomedicine and Prevention, University of Rome Tor Vergata, Rome, Italy; Department of Biomedicine and Prevention, University of Rome Tor Vergata, Rome, Italy; Department of Biomedicine and Prevention, University of Rome Tor Vergata, Rome, Italy; Department of Biomedicine and Prevention, University of Rome Tor Vergata, Rome, Italy; Department of Biomedicine and Prevention, University of Rome Tor Vergata, Rome, Italy; Department of Biomedicine and Prevention, University of Rome Tor Vergata, Rome, Italy; Department of Biomedicine and Prevention, University of Rome Tor Vergata, Rome, Italy; Department of Biomedicine and Prevention, University of Rome Tor Vergata, Rome, Italy; Department of Biomedicine and Prevention, University of Rome Tor Vergata, Rome, Italy; Department of Biomedicine and Prevention, University of Rome Tor Vergata, Rome, Italy

## Abstract

Spontaneous coronary artery dissection (SCAD) is the most common cause of myocardial infarction during pregnancy or the postpartum period and has a major impact on cardiovascular morbidity and death in pregnant women. A 38-year-old woman with sudden cardiac arrest ten days postpartum urgently underwent coronarography, which showed an intraparietal hematoma of the left anterior descending (LAD) artery. Two days later, coronary computed tomography angiography (CCTA) was performed, which showed the evidence of SCAD in the mid-distal tract of LAD and the presence of transmural ischemic infarction in the apex and mid antero-septal wall in delayed acquisition. The patient was treated with a beta-blocker. Four months later CCTA showed complete resolution of SCAD and evolution of the infarcted areas. Given the high accuracy and noninvasiveness of CCTA, our case highlights the potential role of this imaging modality in the diagnosis and follow-up of pregnancy associated SCAD.

## INTRODUCTION

The term spontaneous coronary artery dissection (SCAD) refers to the sudden appearance of a false lumen within the coronary artery caused by either an intimal tear or abrupt hemorrhage within the tunica media of the arterial wall and unrelated to trauma or iatrogenic damage. Acute myocardial infarction (MI) and cardiogenic shock are the main consequences of SCAD [1, 2].

SCAD primarily affects younger women, with a prevalence of 35% in women under 50 years of age and 43% in pregnancy-associated myocardial infarction (PAMI) [3].

Nevertheless, SCAD is often misdiagnosed in the absence of cardiac risk factors because women are generally not at high risk for developing cardiovascular disease. Given the lack of risk indicators, early diagnosis is essential for appropriate patient care [4].

## CASE REPORT

**Figure 1:**
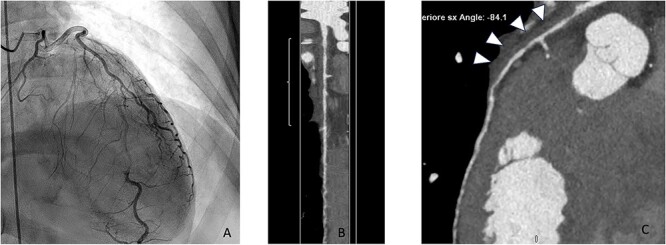
Coronary angiography (June 2023) showing an abrupt change in vessel caliber at the middle-proximal segment of LDA with a long smooth tubular lesion due to intramural hematoma with no visible dissection plane. The caliber of the downstream tract of LDA and the remaining coronary vessels is preserved (**A**). At CCTA post-processing, lumen (**B**) and curved (**C**) reconstructions confirm a jump in caliber at the mid-proximal segment of LDA affected by a crescentic hypodense wall thickening (white arrowheads) compatible with non-supplied intramural hematoma.

In May 2023, a 38-year-old woman with recent onset of peripartum hypertension presented to our emergency department ten days postpartum after having been intubated and treated with prolonged massage and direct current shocks for cardiac arrest with ventricular fibrillation in another hospital. She had neither risk factors nor a family history of cardiovascular events. We performed a cerebral CT angiography showing bilateral dissection of the internal carotid arteries in the mid-extracranial segment with good patency of the distal vessel lumen. Our interventional radiologist gave no indication for interventional treatment. The patient underwent coronary angiography, which showed no significant coronary stenosis but the presence of an intramural hematoma in the mid-distal tract of LAD (Fig. 1A). The patient was treated conservatively with heparin therapy, dual anti-aggregation and an intravenous beta-blocker. Intravascular ultrasound (IVUS), which could have confirmed the diagnosis of SCAD, was not performed no to expose the patient to additional risks. Two days later, the patient underwent a CCTA in our radiology department, since it is a rapid, low-dose examination requiring little contrast medium providing information on coronary arteries, myocardial tissue and cardiac function at the same time.

**Figure 2:**
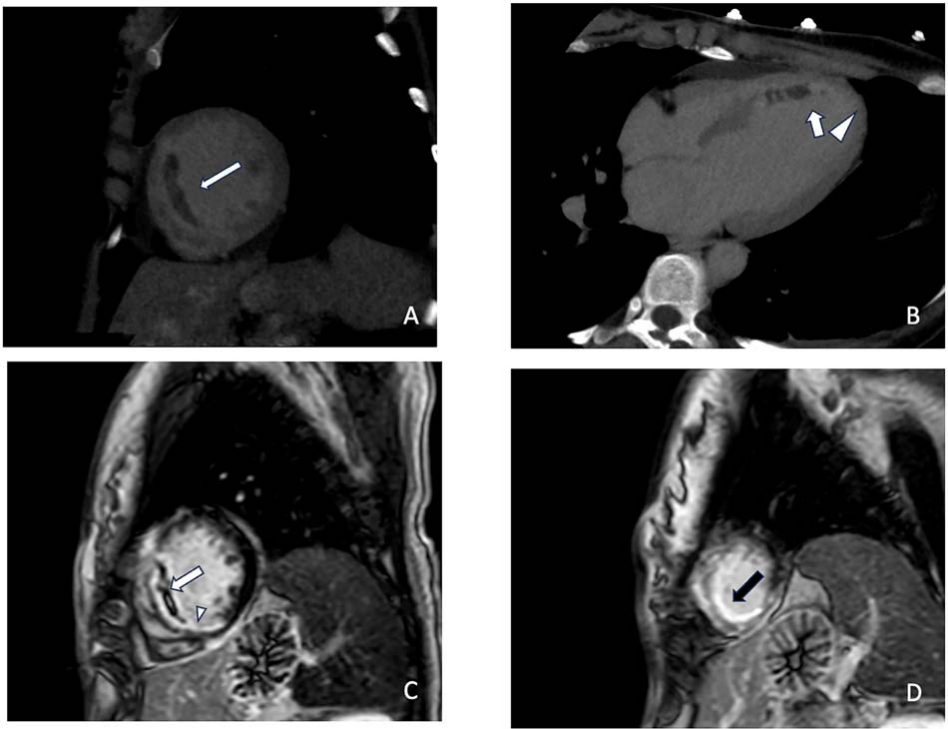
June 2023 LIE-CT scan on short-axis (**A**) and four-chamber (**B**) reconstructions show areas of LIE in the mid-septal (white arrow in **A** and **B**) and apical segment (white arrowhead in **B**). Note the hypodensity area included in the LIE of the septal segment compatible with MVO. One week later, CMR confirmed superimposable extension of the infarcted areas with LGE clearly visible on short-axis (**C**, white arrow) and long-axis four-chamber (**D**, black arrow). MVO: microvascular obstruction.

**Figure 3:**
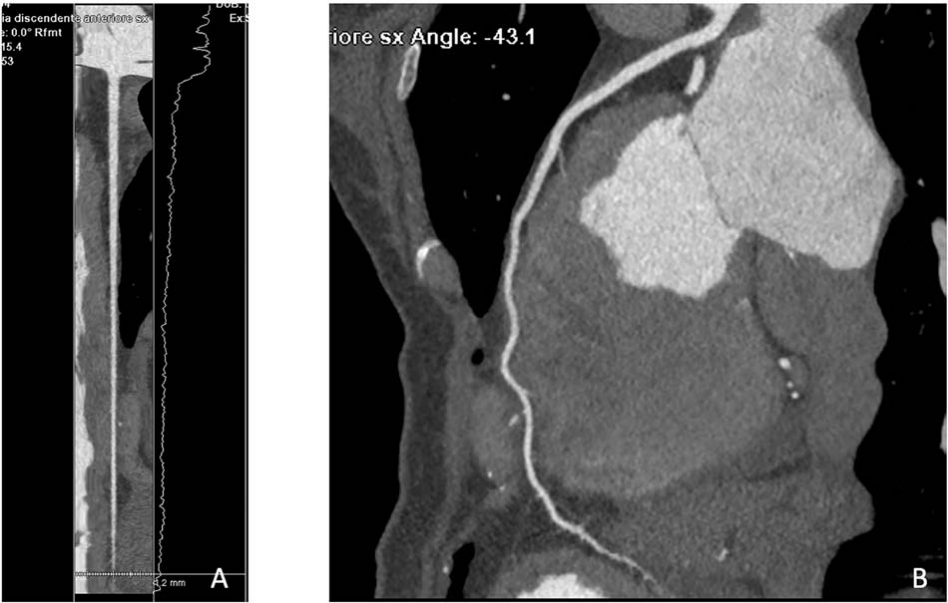
In august 2023 CCTA lumen (**A**) and curved (**B**) reconstructions show resolution of the intramural hematoma at the mid-proximal tract of LDA with restoration of regular vessel caliber.

**Figure 4:**
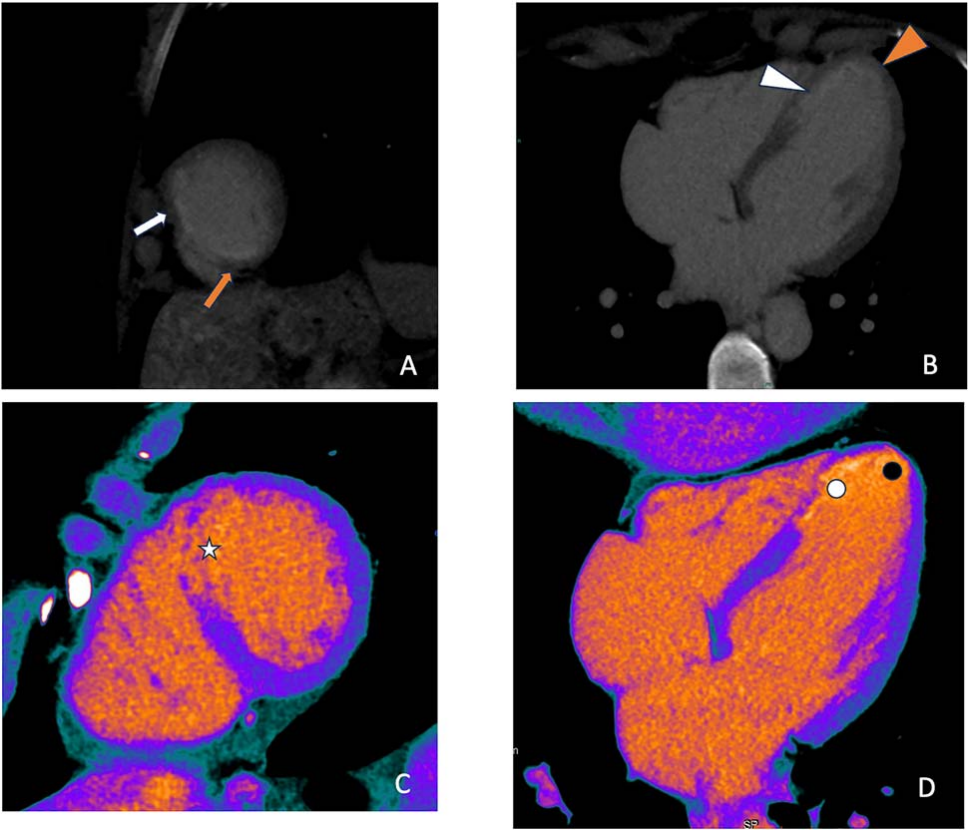
Delayed scan at follow-up CT in august 2023 shows nearly transmural LIE of the mid-septal, apical-septal and apex on short axis (**A**, white and red arrow) and four chamber (**B**, white and red arrowheads) reconstructions with MVO no longer appreciable. The same findings are more easily visualized on colorimetric perfusion maps (short axis in **C** with white star; four-chamber view in **D** with white and black point).

CCTA was performed with a 512-slice CT (GE-Healthcare CT Revolution System, General Electric, Milwaukee, WI, USA) acquiring a first angiographic phase with a retrospective ECG-gating and a second delayed phase 8 min after the injection of contrast media to evaluate the myocardium. CCTA confirmed the presence of an intraparietal hematoma in the mid-distal tract of LAD with good patency of the vessel distally (Fig. 1B and C), while the late iodine enhancement (LIE) scan showed the presence of an extensive transmural LIE in the apex and mid-apical and mid-septal segments (Fig. 2A and B). Thanks to the retrospective gating, we could evaluate ventricular kinetics and function using a semiautomated post-processing software (Adw. 4.7 GE—General Electric, Milwaukee, WI, USA) which documented an ejection fraction of 35% and akinesia of the apex and interventricular septum (IVS). A week later, the patient’s clinical conditions improved and a CMR was performed, confirming akinesia of the apex and IVS and left ventricular EF of 40% associated with an extensive area of late gadolinium enhancement (LGE) in the apex and mid-septal segments (Fig. 2C and D) overlapping the LIE documented on the previous CT examination. One month after her admission, the patient was discharged asymptomatic and in good hemodynamic compensation in terms of further clinical course, which was free of complications and serious arrhythmic events. In September, at four months follow-up, CCTA showed resolution of LAD dissection (Fig. 3A and B) with recovery of LVEF at 50% despite persistent apical akinesia and apical and septal LIE (Fig. 4).

## DISCUSSION

SCAD usually affects young women who have few or no risk factors for atherosclerosis. Clinically, it manifests as acute coronary syndrome (ACS), which is often poorly suspected in these patients, leading to possible misdiagnosis [1]. It causes 35% of MI in women younger than 50 years and 43% of MI occurring in the peri- or postpartum period [3].

Hemodynamic and hormonal changes of the peri-partum period can increase vessel wall stress through the development of eosinophilic infiltrate. Following wall damage, intramural hematoma and false lumen formation occur through micro-infiltration of blood into the intima and rupture of vasa vasorum. Although there is still no consensus on the best treatment for SCAD, conservative treatment with aspirin and beta-blockers is associated with good in-hospital outcome, whereas percutaneous coronary intervention (PCI) may lead to dissection propagation and coronary artery by-pass graft (CABG) may fail because of competing flow from the native healed artery [5, 6].

The gold standard imaging modality for patients with ACS is coronary angiography, which can be aided by IVUS in uncertain cases [1]. CMR with late gadolinium enhancement (LGE) is an indispensable tool for noninvasive assessment of SCAD-induced complications, characterization of myocardial structure, detection of typical ischemia in the area of suspected dissection and evaluation of global or segmental myocardial contractility abnormalities [7, 8]. Therefore, considering that LGE is a predictor of poor cardiovascular outcome, myocardial scarring must be accurately and rapidly identified to diagnose infarction and select the most effective treatment for each patient. However, despite the undeniable clinical importance of CMR, it is not widely used in acute care settings, mainly because patients with ACS, including those with SCAD, are often hemodynamically unstable and cannot tolerate long scan times [7].

Recently CCTA has become a tool to evaluate not only coronary arteries, but also myocardial tissue and cardiac funtion [7, 9]. Iodinated contrast media have different concentrations between healthy and scar tissue, the latter of which can be identified as areas of LIE at 10–15 min scan [7, 10].

In our case, CCTA results matched both coronarography and CMR findings. Using a rapid and noninvasive examination, we confirmed the diagnosis of SCAD, evaluated myocardial damage and assessed ventricular function.

Considering the frequent association between postpartum SCAD and carotid artery dissection, CT may be the best imaging technique for proper diagnostic evaluation of these patients. Indeed, with a single rapid and readily available CT scan combining a retrospective ECG-gated acquisition with a volumetric acquisition of the epiaortic vessels, as well as a late acquisition to evaluate LIE of the myocardium, we are able to provide all the information needed for correct diagnostic and prognostic evaluation.
